# Enhanced Diagnosis of Lung and Colon Cancer Severity Through Deep Feature Analysis Using DenseNet201 and SVM With Histopathological Images: A Super‐Resolution Approach

**DOI:** 10.1002/cnr2.70439

**Published:** 2025-12-23

**Authors:** Pragati Patharia, B. Surya Prasad Rao, Prabira Kumar Sethy, Ashoka Kumar Ratha, Aziz Nanthaamornphong

**Affiliations:** ^1^ Department of ECE Guru Ghasidas Vishwavidyalaya Bilaspur India; ^2^ PVP Siddhartha Institute of Technology Vijaywada India; ^3^ Department of Electronics Sambalpur University Sambalpur Odisha India; ^4^ College of Computing Prince of Songkla University Phuket Thailand

**Keywords:** colon cancer, DenseNet201, FSRCNN, histopathology images, lung cancer, medical imaging, SVM

## Abstract

**Background and Aim:**

The worldwide healthcare system faces significant challenges due to the increasing prevalence of lung and colon cancer, highlighting the need for timely and accurate detection to improve patient prognosis. The precision of cancer diagnosis is highly dependent on the expertise of histopathologists, making it a complex and demanding endeavor. A shortage of sufficiently skilled experts can lead to the ineffective allocation of healthcare resources, potential misdiagnoses, and unwarranted interventions, ultimately threatening patient well‐being. However, technological advancements have introduced deep learning as a powerful tool in clinical applications, particularly in the field of medical imaging. This study aims to develop a novel diagnostic method leveraging deep learning techniques to enhance the accuracy of lung and colon cancer detection.

**Methods:**

The study utilized the LC25000 dataset, which comprises 25 000 histopathological images of lung and colon tissue. A novel approach was implemented using a Fast Super‐Resolution Convolutional Neural Network (FSRCNN) for image enhancement and a Support Vector Machine (SVM) model based on DenseNet201 for classification. The FSRCNN was employed to improve the clarity and detail of images by increasing their resolution, which is crucial for accurate cancer detection.

**Results:**

The proposed model demonstrated superior performance compared to existing Convolutional Neural Network (CNN) models. It achieved an overall accuracy of 98.00%, precision of 98.10%, sensitivity of 98%, F1 score of 0.98, and specificity of 99.50%. These metrics indicate a significant enhancement in diagnostic accuracy and reliability, underscoring the effectiveness of the FSRCNN and SVM‐based DenseNet201 model.

**Conclusion:**

The implementation of the FSRCNN and SVM‐based DenseNet201 model provided substantial improvements in the detection of lung and colon cancer, as evidenced by high accuracy and specificity metrics. While the LC25000 dataset offered a solid foundation for this analysis, future research should aim to validate the model's effectiveness using a broader array of diverse and extensive datasets. Additionally, integrating supplementary diagnostic techniques, such as genetic data and electronic health records, could further enhance the model's diagnostic precision and practical application in cancer diagnosis.

## 1 Introduction

The global burden of cancer‐related illness and mortality is significantly influenced by lung and colon cancer. On an annual basis, lung cancer results in approximately 1.76 million fatalities and 2.06 million new cases, while colorectal cancer causes 783 000 fatalities and 1.80 million new cases [1]. It is essential to conduct a precise and timely diagnosis of the histology of lung cancer to determine the most appropriate treatment method, as the therapeutic choices are influenced by the specific subtypes of the disease, its stage, and its molecular characteristics [2]. “Tobacco use, alcohol consumption, obesity, dietary variables, and infections are among the primary risk factors associated with the development of lung and colon malignancies [3].” “To mitigate the adverse effects of cancer and increase the likelihood of survival, it is crucial to implement reliable screening procedures that can promptly identify malignant conditions. Technological advancements have significantly transformed the approach to cancer diagnosis and treatment. In national lung cancer screening programs, low‐dose computed tomography (CT) imaging has shown significant potential for reducing mortality rates [4].” In addition, the utilization of artificial intelligence (AI) and ML techniques has become increasingly prevalent in expediting cancer diagnosis. This enables the screening of large groups of patients at a faster pace and lower cost [5]. DL methods have shown significant potential for enhancing the precision and efficacy of cancer categorizations based on histological images, which has emerged as a critical field of research. Histopathological examination is critical in cancer diagnosis and provides essential information regarding the structure of cells and tissues. However, conventional methods frequently require tedious manual evaluation by pathologists, which leads to subjective results and time delays. CNNs, specifically DL, have emerged as an effective approach for automating cancer categorization in response to these obstacles. These models have demonstrated exceptional proficiency in the accurate identification of intricate patterns and features, enabling them to detect cancer using medical imaging [6, 7]. The early identification of lung and colon malignancies is a significant potential application of DL‐ and ML‐based image‐processing algorithms. These methods exhibit exceptional proficiency in the analysis of histological images to distinguish between benign and malignant illnesses, classify cancer types and stages, and acquire knowledge from extensive datasets without the need for human intervention or preexisting knowledge. Compared with conventional diagnostic methods, DL models have consistently exhibited superior performance in terms of resilience, specificity, sensitivity, and accuracy [8]. Furthermore, image‐processing techniques enhance the quality of images, reduce noise, identify specific regions of interest, extract characteristics, and perform data augmentation. These enhancements have a substantial impact on the advancement of cancer diagnostics [9]. The analysis of medical images is extremely suitable for DL models, specifically CNNs, which are a powerful option for detecting lung and colon malignancies [10]. These models demonstrate extraordinary sensitivity and specificity in the identification of anomalies in the lung and colon tissues, ensuring precise differentiation between healthy and malignant tissues. This ability is essential for timely diagnosis and intervention of medical conditions. “The LC25000 dataset has been employed to develop a computer‐assisted diagnosis technique that accurately detects lung and colon tumors using CNNs [11, 12].” “Three CNN architectures were implemented: ResNet‐30, ResNet‐18, and ResNet‐50. ResNet‐50 exhibited a maximum accuracy of 93.91% among these models, with ResNet‐30 and ResNet‐18 closely following in their wake [13].” Nevertheless, the sector's ongoing challenge is to enhance and perfect automated diagnostic systems, despite the progress achieved thus far.

A DL‐based classification framework is employed by Masud et al. to diagnose lung and colon cancer using a ML technique. “This groundbreaking study demonstrated the ability of DL techniques to improve the precision of cancer diagnosis, specifically in the classification of histopathological images [14].” Al‐Jabbar et al. made strides in this field by employing histopathological analysis to identify lung and colon cancer malignancies through hybrid systems that integrated various feature modalities. Their hybrid approach underscores the benefits of integrating a variety of diagnostic modalities to enhance the precision and reliability of cancer classification models [15]. Fan et al. introduced a transfer learning framework that incorporates a SVM to classify histopathology images. “This investigation underscores the importance of transfer learning in optimizing model performance, particularly in scenarios where annotated data are scarce [16].” Mehmood et al. improved this method by concentrating on the identification of malignant cells in histopathology images of the lung and colon. “This was accomplished by integrating transfer learning with class‐selective image processing techniques. Their research emphasized the adaptability of pre‐trained models in resolving specific cancer classification issues, thereby demonstrating the advantages of transfer learning in improving diagnostic precision [17]”. A novel method for the detection of lung and colon cancers was introduced by Omar et al. [18]. “This method incorporates the use of a weighted average ensemble transfer‐learning technique. Our strategy demonstrated the potential of ensemble techniques to improve the overall accuracy and reliability of cancer diagnosis systems by integrating numerous models, thereby improving the classification performance [18].”

An investigation into the detection of colon cancer was conducted by Sakr et al. [19]. “They proposed a highly effective DL method and emphasized the importance of optimizing DL models for both computational economy and accuracy. The objective of this study was to improve the accuracy of cancer diagnosis and optimize resource utilization by addressing the computational challenges associated with the process [19].” Yehia et al. proposed a method to enhance the detection of lung and colon cancer in their study. “They employed a combination of DL techniques and double‐contrast limited adaptive histogram equalization (CLAHE). Their research underscored the necessity of advanced pre‐processing methods to improve the diagnostic capabilities of DL models, particularly in the areas of image quality and contrast [20].” Attallah et al. introduced an exhaustive framework for the diagnosis of lung and colon cancer. “This system incorporates transformation techniques with efficient deep learning (DL) models. This method underscores the potential of utilizing efficient architectures to achieve precision diagnostic results while reducing the computational burden [21].” Uddin et al. proposed two dense architectures, D1 and D2, designed to enhance colon and lung cancer image classification, demonstrating superior accuracy and resilience across multiple datasets, including NCT‐CRC‐HE‐100K, CRC‐VAL‐HE‐7K, LC25000, and IQ‐OTHNCCD. D1 achieved 99.80% accuracy on NCT‐CRC‐HE‐100K, while an ensemble of D1 and D2 reached 93% on IQ‐OTHNCCD, outperforming existing models even with imbalanced data. The models proved robust under limited training data, and D2 exceeded benchmarks like InceptionV3 and DenseNet201 with a 96% accuracy on CRC‐VAL‐HE‐7K. Additionally, explainable AI tools such as Grad‐CAM were used to visualize key features, emphasizing the models' promise for improving early cancer diagnosis and global healthcare access [22]. Nabeel et al. developed a machine learning approach to improve the precision of lung cancer detection, addressing the need for more accurate, less invasive, and cost‐effective diagnostic techniques. By benchmarking four methods against established techniques on a Kaggle lung cancer dataset, one model emerged as particularly effective, achieving a remarkable accuracy of 99.16%, precision of 98%, and sensitivity of 100%. Key to this model's success was hyperparameter tuning, specifically adjusting the Gamma and C values to 10, optimizing kernel width and regularization. This approach demonstrates significant improvements over existing diagnostic strategies for lung cancer detection [23]. Li et al. developed a hybrid feature extraction approach for lung cancer detection, combining Gray‐level co‐occurrence matrix (GLCM) with Haralick features and autoencoder‐based features. These features were used with supervised machine learning models, where SVM with Radial Base Function (RBF) and Gaussian kernels achieved near‐perfect performance, while SVM polynomial reached an impressive accuracy of 99.89% when using GLCM, Haralick, and autoencoder features. SVM Gaussian and SVM RBF also performed strongly with accuracies of 99.56% and 99.35%, respectively, using GLCM with Haralick features. This approach shows promise for enhancing diagnostic accuracy in lung cancer treatment and decision‐making [24]. Yang et al. conducted a study on lung cancer diagnosis, focusing on detailed posterior probability analysis to explore associations among Gray‐level co‐occurrence matrix (GLCM) features. Using a t‐test for feature ranking, they identified Cluster Prominence as a key feature and analyzed its associations using mutual information. When Cluster Prominence was at a state ≤ 330.85, the model achieved a perfect ROC index (100%) and a relative Gini index of 99.98%. This method provides a deeper understanding of GLCM feature dynamics, enhancing lung cancer diagnosis and prognosis accuracy [25].

Table 1 provides a summary of the literature review on the classification of lung and colon cancer. This review focuses on the application of numerous CNNs to the classification of malignancies using medical images. CNNs independently acquire essential characteristics from medical images, resulting in precise tumor detection. To train the classifiers, conventional ML methods extract features from images, including texture, shape, and intensity. In addition, the integration of clinical information, including patient medical history and demographics, with imaging data has improved the precision of cancer classification models.

**TABLE 1:** A thorough analysis of studies on the detection and classification of lung and colon cancer with the LC25000 dataset.

References	Methods	Results	Limitations
Masud et al. [14]	A DL and digital image processing classification system	Diagnosed malignant lung and colon tissues with 96.33% accuracy.	The study classifies lung and colon tissues using DL, although it does not explore clinical challenges. No mention is made of the computational resources and time required to train and deploy the classification system.
Al‐Jabbar et al. [15]	First, artificial neural networks (ANN) containing key GoogLeNet and VGG‐19 characteristics were applied.	ANN obtained 96.49% sensitivity, 96.19% precision, 95.50% accuracy.	The study employs transfer learning with class‐selective image processing to identify malignancy in lung and colon histopathology pictures, which could limit its applicability to other malignancies or medical imaging tasks.
Fan et al. [16]	Compares the standard SoftMax classifier with the transfer learning strategy.	Cross‐validation accuracy was over 0.99 for all four folds, averaging 0.9929.	The study does not examine the benefits and drawbacks of combining the SVM classifier with the softmax‐based model's fully‐connected layer, which might affect performance.
Mehmood et al. [17]	Use the AlexNet pretrained model from the ImageNet competition to categorize cancer images	Model accuracy from 89% to 98.4%	The analysis highlights those numerous datasets with different scan settings might provide false‐positive findings, suggesting dataset variability is limited.
Omar et al. [18]	A combined transfer learning approach that enhances the performance of VGG16, Inception V3, and MobileNet V1 models.	VGG16, Inception V3, and MobileNet V1 have accuracy of 96.93%, 98%, and 98.32% respectively.	Lack of comparison with other state‐of‐the‐art cancer detection models or methodologies, which might improve the ensemble transfer learning model's assessment.
Sakr et al. [19]	A lightweight CNN‐based DL method.	The suggested DL method detected colon cancer with 99.50% accuracy.	The study does not cover implementation or testing challenges, which could demonstrate the approach's effectiveness.
Yehia et al. [20]	DL to diagnose preprocessing with enhancement, EfficientNetB7, and Modified Neural Network (MNN) stages.	Training and testing dataset performance and achieving 99.5% classification accuracy.	The study's Double CLAHE and DL approach's attainable drawbacks are not addressed.
Attallah et al. [21]	The framework uses lightweight DL models ShuffleNet, MobileNet, and SqueezeNet for efficient processing.	Lightweight DL model framework distinguished lung and colon cancer variants with 99.6% accuracy.	The study did not compare lightweight DL models to resource‐intensive models to evaluate accuracy and computational complexity trade‐offs.
Uddin et al. [22]	Proposed two dense architectures, D1 and D2, for colon and lung cancer image classification; used datasets like NCT‐CRC‐HE‐100K, CRC‐VAL‐HE‐7K, LC25000, IQ‐OTHNCCD; incorporated explainable AI tools (e.g., Grad‐CAM).	D1 achieved 99.80% accuracy on NCT‐CRC‐HE‐100K; ensemble of D1 and D2 reached 93% accuracy on IQ‐OTHNCCD; D2 achieved 96% accuracy on CRC‐VAL‐HE‐7K.	May require further testing on additional cancer types and various imaging modalities.
Nabeel et al. [23]	Developed ML models with hyperparameter tuning (Gamma and C set to 10) for lung cancer detection; benchmarked against standard techniques on Kaggle lung cancer dataset.	Achieved 99.16% accuracy, 98% precision, and 100% sensitivity, demonstrating significant improvement over existing diagnostic techniques.	Limited to Kaggle dataset; needs validation across larger and more diverse datasets.
Li et al. [24]	Hybrid feature extraction combining GLCM with Haralick features and autoencoder‐based features; used SVM models (RBF, Gaussian, Polynomial) for Lung cancer detection	SVM polynomial achieved 99.89% accuracy; SVM Gaussian and SVM RBF achieved 99.56% and 99.35%, respectively, showing potential for enhancing diagnostic accuracy.	Potentially computationally intensive due to hybrid extraction; may need optimization for real‐time use.
Yang et al. [25]	Posterior probability analysis on GLCM features for lung cancer; ranked features using t‐test, selected Cluster Prominence as target feature, assessed mutual information.	Achieved 100% ROC index and 99.98% relative Gini index, offering deeper insights into GLCM feature dynamics and associations.	Primarily limited to GLCM‐based features; additional feature sets could improve versatility and accuracy.

A synopsis of the literature provides a thorough examination of several DL and digital image processing methods used in the identification of cancer, with a primary emphasis on lung and colon cancer. The experiments used the LC25000 dataset, which consisted of 25 000 photos for training and testing purposes. Various techniques, such as CNN, transfer learning, ensemble models, and lightweight DL architectures, have been used to accurately categorize cancer tissues. In general, this research emphasizes the efficacy of DL methods in the successful diagnosis of cancer based on histopathological images. Therefore, DL has the potential to significantly improve medical diagnostic techniques.

Although these findings add to the growing amount of research on DL‐based cancer classification, there remains a need for effective and precise models that are especially designed to tackle the difficulties related to diagnosing lung and colon cancer. This paper presents an innovative DL framework for classifying lung and colon cancers based on the gaps observed in previous research. We are prompted by the lack of research that addresses the classification of colon cancer using DL methods, despite the existence of large datasets, such as the LC25000 dataset. Our objective was to use extensive DL methods to create accurate and robust models that can effectively identify lung and colon cancers. This will eventually enhance diagnostic skills in the clinical setting. This article summarizes the current advancements in technologies, methodologies, and outcomes that have led to the development of innovative solutions for classification tasks.

The suggested revolutionary deep learning (DL) framework for classifying lung and colon cancers is a significant breakthrough in the application of sophisticated DL techniques to improve diagnostic accuracy and capability. The objective of this study was to introduce an FSRCNN Technique for diagnosing the severity of lung and colon cancers by deep feature analysis using DenseNet201 and SVM. These developments have expanded the limits of performance measurements, thereby improving the effectiveness and accuracy of diagnostic models in the field of cancer categorization.

Overall, this study makes the following key contributions:

- This study introduces a new DL model that combines the DenseNet201 architecture with an SVM classifier, resulting in superior performance for lung and colon cancer diagnosis.
- The use of the FSRCNN significantly improved the resolution and clarity of the histopathological images, contributing to the model's high diagnostic accuracy.
- The model achieved outstanding performance, with an overall accuracy of 98.00%, precision of 98.10%, sensitivity of 98%, F1 score of 0.98, and specificity of 99.50%, thereby proving its effectiveness.
- The DenseNet201‐based SVM model was established as a reliable tool for diagnosing lung and colon cancers, showing stability and consistency across evaluative metrics, with potential for clinical applications.
- This study suggests that future research could improve diagnostic precision by integrating other modalities such as genomic data or electronic health records, setting a foundation for more comprehensive cancer diagnostics.

## 2 Materials and Methodology

### 2.1 About Dataset

The dataset was collected from the Kaggle Repository. “It consists of 25 000 color images, each with a resolution of 768 × 768 pixels, encoded in JPEG format, and systematically organized into five distinct classes, each containing 5000 images. These classes are divided into two main types: colon and lung cancer. The Colon Cancer subset includes two subtypes: colon adenocarcinoma (colon_aca) and benign colon tissue (colon_n). The Lung Cancer subset consists of three subtypes, namely Lung Adenocarcinomas (lung_aca), Lung Squamous Cell Carcinomas (lung_scc), and Benign Lung Tissues (lung_n) [26]”.

**FIGURE 1:**
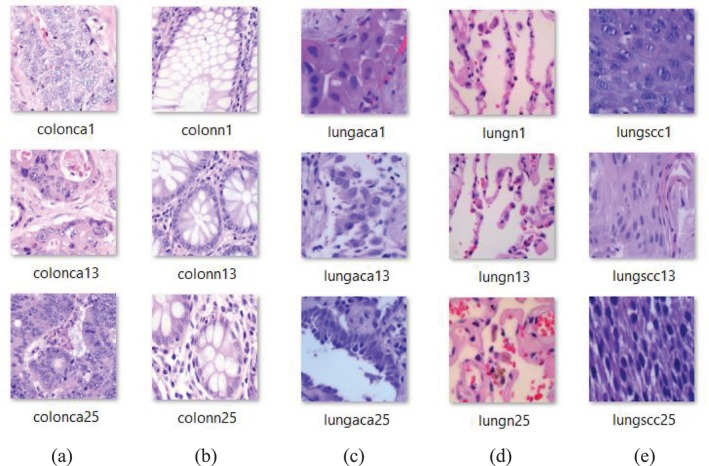
Samples of Histopathological Images (a) Colon_aca, (b) Colon_n, (c) Lung_aca, (d) lung_n, and (e) lung_scc.

Figure 1 illustrates sample images from each class within the dataset, whereas Table 2 provides a detail distribution of dataset.

**TABLE 2:** Categorization of histopathological images.

Histopathological images	Number of images
Colon_aca	5000
Colon_n	5000
Lung_aca	5000
Lung_n	5000
Lung_scc	5000

### 2.2 Super Resolution Technique

Super‐resolution techniques are employed to reconstruct high‐resolution image details from low‐resolution lossy inputs. The advent of DL approaches in super‐resolution has garnered significant research attention in recent years owing to their advanced learning capabilities and robustness to noise. DL‐based super‐resolution methods are predominantly applied in domains such as image restoration, medical imaging, and microscopy. This study provides a comprehensive analysis of DL super‐resolution, focusing on the underlying models and architectures driving advancements in this field.

#### 2.2.1 Deep Learning SR Models Using CNN

Two methods for enhancing image resolution: the traditional Bicubic Interpolation, which estimates pixel values based on surrounding pixels, and the Fast Super Resolution Convolutional Neural Network (FSRCNN), which utilizes deep learning techniques. FSRCNN incorporates operations such as shrinking, mapping, expanding, and deconvolution. While these operations are not training processes in themselves, they are essential components of the model's architecture. Shrinking reduces spatial dimensions, mapping transforms the reduced representation into a feature space, expanding increases dimensions back to the desired output size, and deconvolution refines the feature maps during expansion. Together, these methods allow for effective resolution enhancement, capturing fine details and textures in the images.

**FIGURE 2:**
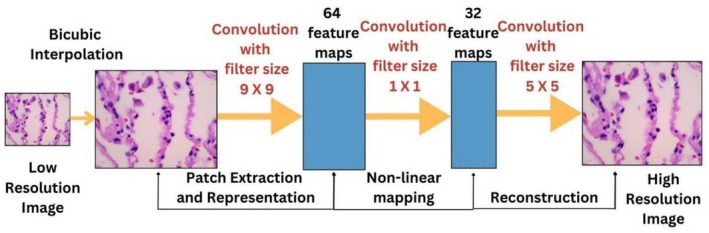
Framework for CNN based on SR.

CNN‐based super‐resolution (SR) models typically employ a fundamental CNN architecture [27], as depicted in Figure 2, incorporating multiple convolutional and pooling layers to extract local salient features from the images. Convolutional layers serve as parallel processing units that capture local features at lower resolutions, thereby facilitating the learning of latent representations. Subsequently, a deconvolution process is employed to upscale the image, restore it to its original size, and generate a higher‐resolution output. This convolution‐deconvolution framework leverages DL techniques and enables the generation of accurate and efficient high‐resolution (HR) images when optimally designed and trained.

Supervised learning approaches are commonly used to train CNN‐based SR models, where a large dataset is utilized to learn mapping from low‐resolution (LR) to high‐resolution images. However, this method presents challenges owing to the necessity of learning a degradation function from training data. Alternatively, unsupervised learning is more suitable for learning degradation functions, as has been explored in various SR model variants.

In the SRCNN, the process begins with bicubic interpolation to upscale the image to the desired resolution. This was followed by a series of convolution operations using 9 × 9, 1 × 1, and 5 × 5 filters to enhance the image quality. The computational complexity of SRCNN is directly proportional to the size of the high‐resolution (HR) image, meaning that larger HR images result in higher complexity.

**FIGURE 3:**
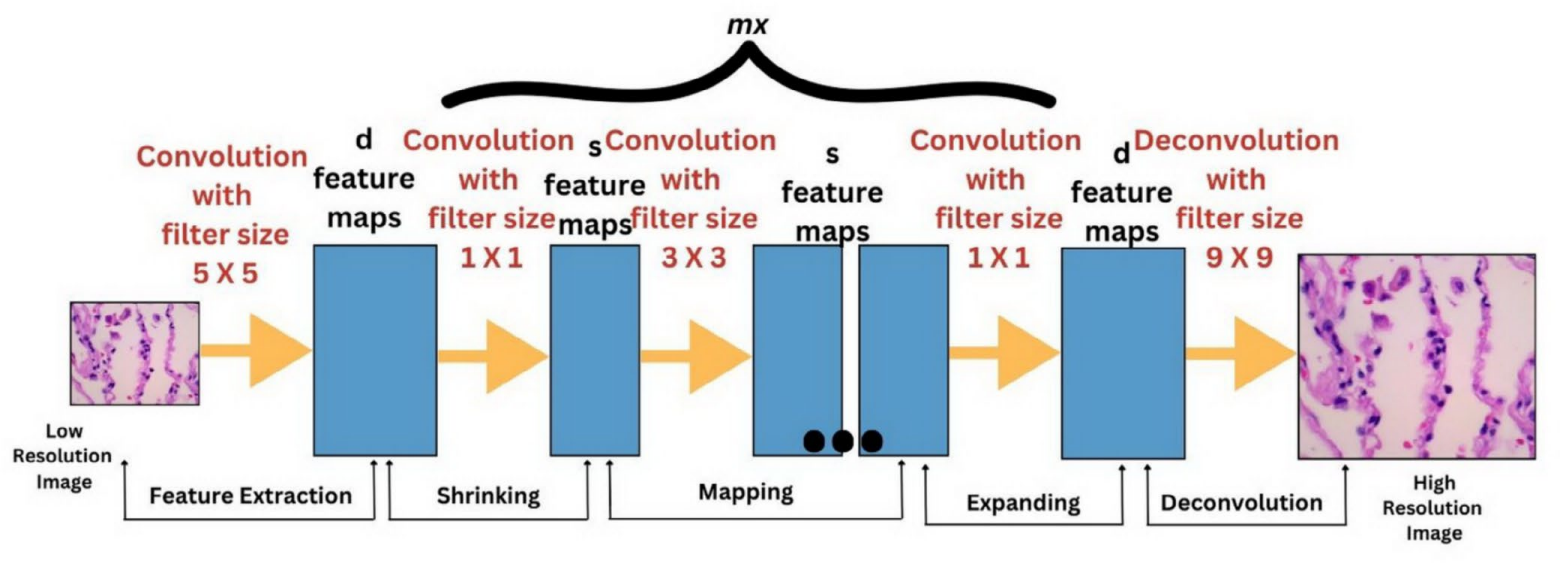
Framework for CNN based on fast SR.

In contrast, the FSRCNN involves five main steps, as shown in Figure 3.

- **Feature Extraction**: The initial bicubic interpolation used in the SRCNN is replaced by a 5 × 5 convolutional layer.
- **Shrinking**: A 1 × 1 convolution was applied to reduce the number of feature maps from *d* to *s*, where *s* was significantly smaller than *d*.
- **Nonlinear Mapping**: Multiple 3 × 3 convolutional layers are utilized instead of a single large layer.
- **Expanding**: Another 1 × 1 convolution is performed to increase the number of feature maps from *s* to *d*.
- **Deconvolution**: Finally, 9 × 9 filters are used to reconstruct the HR image.

In FSRCNN, the computational complexity is directly proportional to the size of the low‐resolution (LR) image, which is significantly lower than that of SRCNN.

**FIGURE 4:**
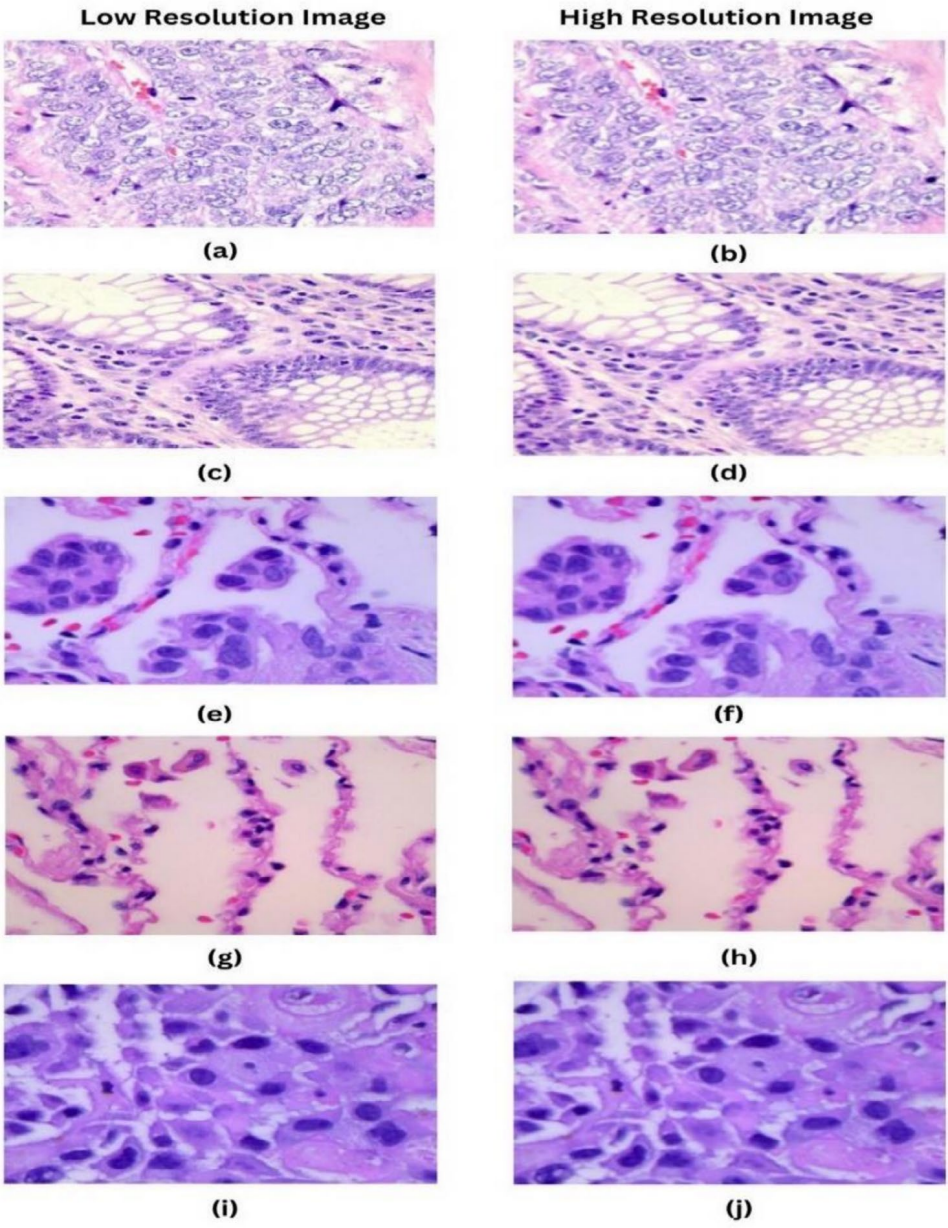
Enhancement of Images using Fast Super‐Resolution CNN method: (a) Original LR image of Colon_aca; (b) Enhanced HR image of Colon_aca; (c) Original LR image of Colon_n; (d) Enhanced HR image of Colon_n; (e) Original LR image of Lung_aca; (f) Enhanced HR image of lung_aca; (g) Original LR image of lung_n; (h) Enhanced HR image of lung_n; (i) Original LR image of lung_scc; (j) Enhanced HR image of lung_scc.

FSRCNN achieves a similar restoration quality to SRCNN, with a significant speed increase, up to 40 times faster, while maintaining efficient computation. Figure 4 illustrates the HR image from the LR image obtained using the FSRCNN technique.

### 2.3 Methodology

**FIGURE 5:**
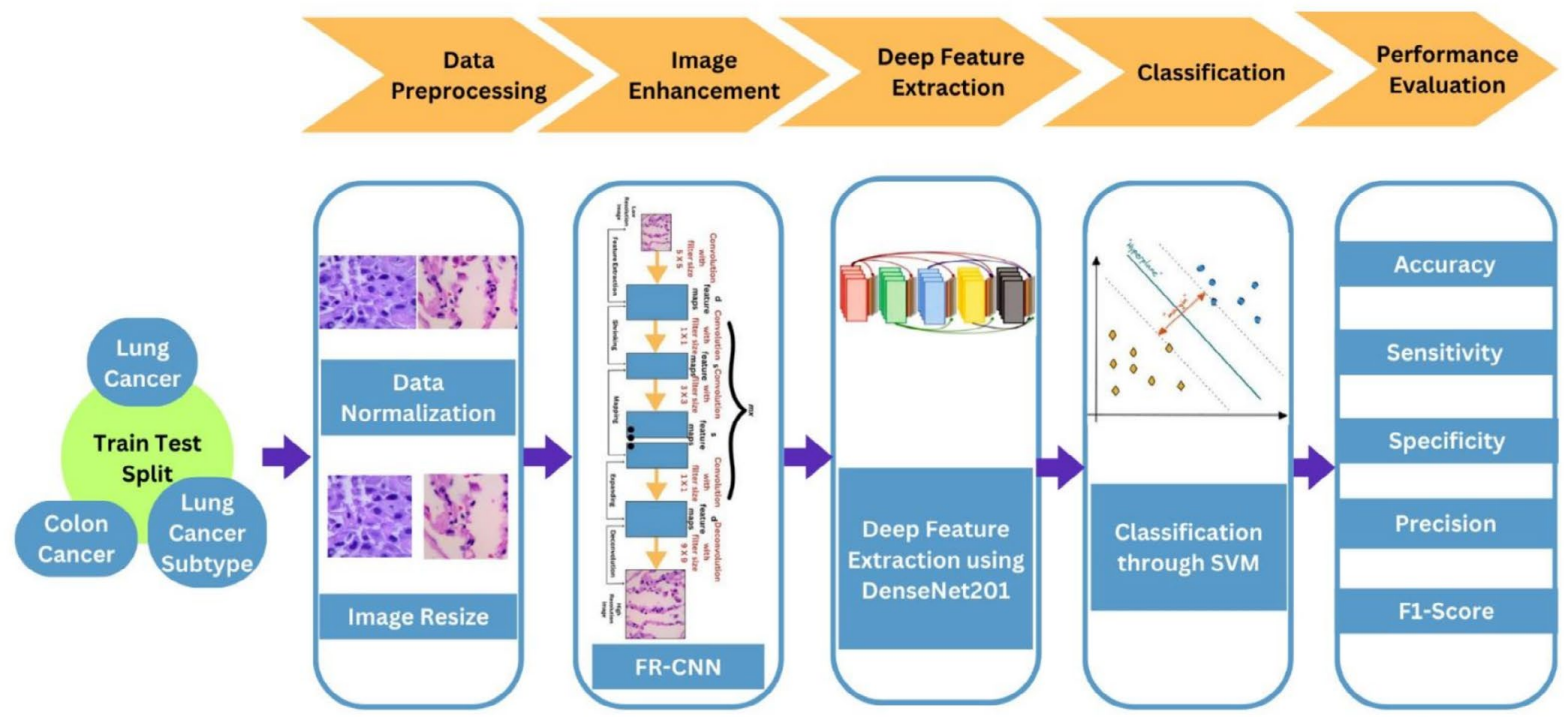
Illustrates the proposed framework.

A novel DL model was developed utilizing the Fast Super‐Resolution CNN (FSRCNN) to classify lung and colon cancer histopathological images. The proposed framework is illustrated in Figure 5. A thorough explanation of the dataset that was used, the preprocessing processes that were implemented, the suggested classification approach, as well as the configuration and training requirements of the model, are provided in the following sections. The framework was structured into three primary components: (1) Data Preprocessing, (2) Deep Feature Extraction and Classification, and (3) Performance Evaluation.

#### 2.3.1 Data Pre‐Processing

Data preprocessing in a CNN that utilizes a pre‐trained model generally involves converting input image into an appropriate form as per model requirement. “This preprocessing typically includes normalizing pixel values to a standardized range, such as [0, 1] or [−1, 1], and applying mean subtraction and standard deviation scaling to ensure consistent input distributions [28].” These procedures are essential for optimizing the model's performance and ensuring that the input data align with the conditions under which the pretrained model was originally trained.

- **Splitting of Data**

The dataset was partitioned into training, validation, and test subsets with allocations of 70%, 20%, and 10%, respectively (Table 3). Each subset was consistently maintained across all the experimental folds, ensuring a uniform distribution of data for the training, validation, and testing phases.

**TABLE 3:** An outline of image distribution within each fold of the dataset.

Histopathological images	Train (70%)	Test (20%)	Validation (10%)
Colon_aca	3501	1000	500
Colon_n	3501	1000	500
Lung_aca	3501	1000	500
Lung_n	3501	1000	500
Lung_scc	3501	1000	500

- **Normalization of Data**

The mean and standard deviation (SD) of all images in the dataset were calculated as part of the preprocessing procedure. Initially, each image was converted to the RGB format, and its pixel values were normalized to a range between 0 and 1. This normalization process involves computing the mean and variance of the pixel values with the standard deviation obtained as the square root of the variance. These statistics were then used to standardize the pixel intensities. To minimize computational complexity, pixel values are scaled by dividing them by 255, thus adjusting them to the 0–1 range. This normalization procedure effectively standardizes the pixel intensity levels across the dataset [29].

#### 2.3.2 Proposed DenseNet201 Architecture

DenseNet architectures, derived from ResNet, optimize the gradient flow and improve the performance with fewer parameters than traditional convolutional networks. In DenseNet, each layer connects to all the preceding layers, promoting efficient feature reuse and simplifying network training. This dense connectivity enhances the information flow and dataset organization. DenseNet201, a variant of this architecture, offers high parameter efficiency by reusing features across layers, increasing the input variability, and boosting the performance. The condensed structure of DenseNet201 reduces the number of parameters, making it particularly effective for DL tasks that require efficient model training.

DenseNet201 has demonstrated exceptional performance on various datasets using pretrained weights owing to its architecture that connects each layer directly to all preceding and subsequent layers, enhancing overall connectivity. This model architecture, which is applied in disease prediction tasks, features three transition layers and four dense blocks. Each dense block utilizes convolutional kernels of sizes 1 × 1 and 3 × 3, repeated 6, 12, 24, and 6 times across the blocks. Transition layers between dense blocks consist of convolution, batch normalization, and pooling layers with a kernel size of 1 × 1, whereas the pooling layer operates with a 2 × 2 stride. The feedforward connections between the convolution layers in dense blocks allow for efficient feature extraction and reuse, contributing to the superior performance of the model.

In the DenseNet architecture, the layers are connected such that each layer receives the feature maps of all the preceding layers as inputs. This can be mathematically described as follows:

The basic formulation for a layer's output in the DenseNet architecture is given by.

(1) $$x_{n}=h_{n}\left(x_{n}-1\right)$$

**FIGURE 6:**
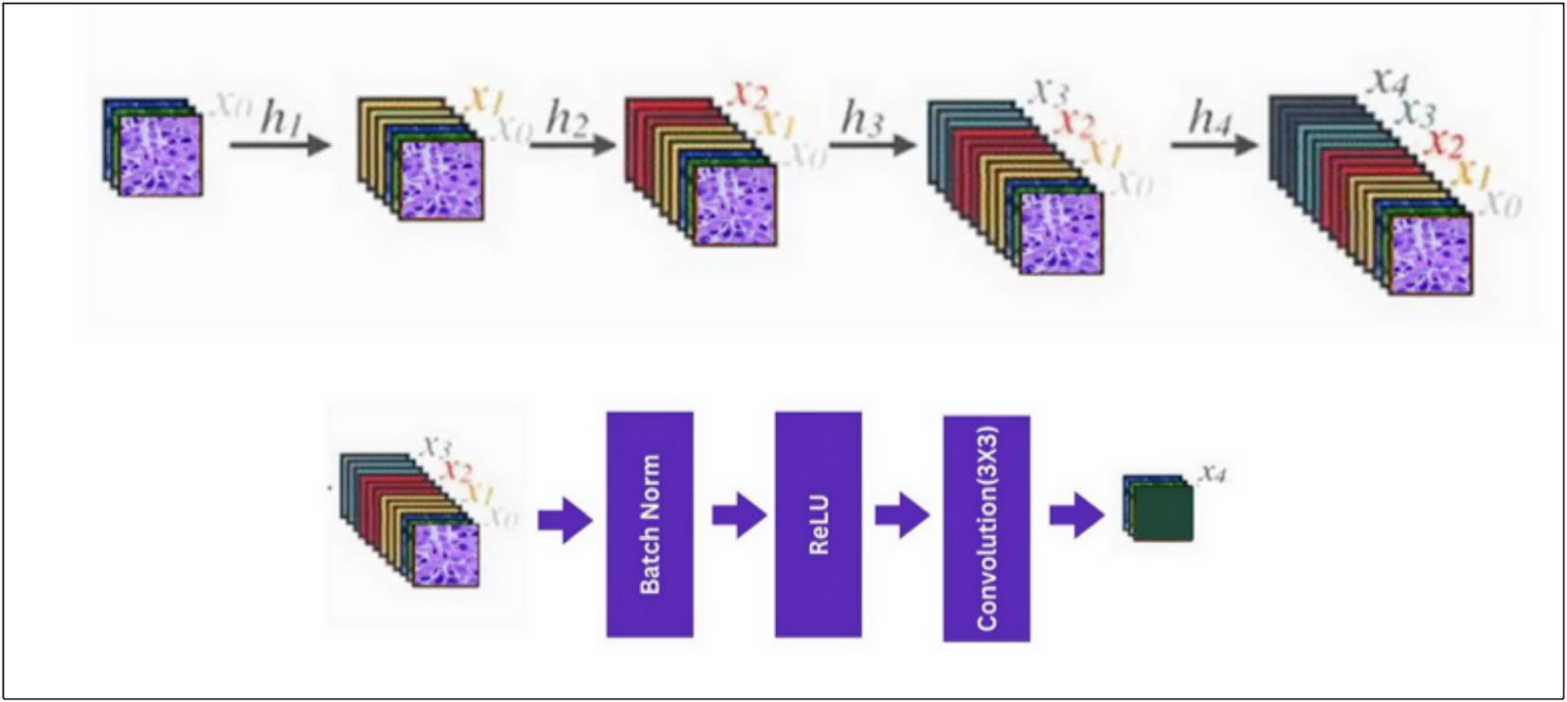
Composition layer.

Here, $x_{n}$ is the output of the n‐th layer, and $h_{n}$ represents the transformation applied at layer n, such as convolution, batch normalization, and activation as shown in Figure 6.

However, unlike traditional architectures, DenseNet combines inputs from all the previous layers without aggregating them. Therefore, Equation (1) is reformulated to account for the concatenation of the feature maps from all preceding layers, resulting in:

(2) $$x_{n}=H_{n}\left(\left[x_{0},\ldots ,x_{n-1}\right]\right)$$

In this formulation, [$x_{0}$,…,$x_{n-1}$] denotes the concatenation of the feature maps from layers 0 through *n* − 1, which is used as input to the current layer $H_{n}$. This structure promotes feature reuse and an efficient gradient flow throughout the network. The proposed DenseNet201 model was designed to accept input images of dimensions 224 × 224 × 3, corresponding to the height, width, and color channels, respectively. The model architecture sequentially employs the following layers: max pooling, dropout, flattening, batch normalization, dense layers, rectified linear unit (ReLU) activation, and fully connected layers. A 3 × 3 kernel was utilized in the max‐pooling layer. To mitigate overfitting, a dropout rate of 0.9 was applied to the dropout layers, effectively deactivating 90% of the neurons. Other dropout rates such as 0.1, 0.3, 0.5, and 0.7 were also tested; however, the 0.9 dropout rate resulted in the best performance and suited the architecture well. Dense layers with 512 neurons were utilized, and batch normalization was performed with the axis value set to −1, ensuring that each feature was normalized. The ReLU activation function, which provides faster computation than the sigmoid and Tanh functions in CNN models, is added prior to the dropout layers. Additionally, a dropout layer 0.9 dropout rate is introduced before the classification layer to prevent overfitting and ensure that the network did not memorize the training data.

#### 2.3.3 Extraction of Deep Features

Deep feature extraction from the fully connected layer of DenseNet201 involves utilizing the learned high‐level representations generated by a dense network. After passing through the dense blocks and transition layers, the features were further refined and integrated into a fully connected layer. This layer consolidates the information extracted from previous layers, enabling the model to capture complex patterns within the input data. These deep features can then be used for various tasks, such as classification, detection, and further analysis, because they represent the most abstract and discriminative characteristics learned by the DenseNet201 architecture.

#### 2.3.4 Colon and Lung Cancer Classification

In the classification of colon and lung cancers, deep features extracted from the fully connected layer of the DenseNet201 model serve as a critical input for a Support Vector Machine (SVM) classifier. DenseNet201, known for its efficient feature extraction capabilities, processes input medical images through a complex network of dense blocks and transition layers. The fully connected layer of DenseNet201 captures high‐level abstract features that effectively represent intricate patterns and characteristics pertinent to cancerous tissues.

These deep features, which encapsulate the nuanced details learned by DenseNet201, were then fed into an SVM classifier. SVM is employed to create a decision boundary in the feature space, enabling precise classification between colon and lung cancers. By leveraging the dense network's powerful feature extraction with SVM's robust classification mechanism, this approach enhances the accuracy and reliability of cancer diagnosis. This combination harnesses the strengths of both models, ensuring effective discrimination between different cancer types based on the deep informative features extracted by DenseNet201.

## 3 Experimental Studies

Empirical evaluations of the proposed DL model were carried out using MATLAB software on a system configured with a Core i5 processor running at 2.50 GHz and supported by an NVIDIA GTX3050 graphics card. This configuration, coupled with 16GB of RAM, provided adequate computational resources for analyzing the classification results of both colon and lung cancers as generated by the algorithm.

The research results are discussed in the following subsections. First, HR histopathological images of both colon and lung cancers were employed to assess the performance of the suggested DL model. Next, the accuracy metrics achieved by the DL model were presented. Finally, the performance of the proposed model was compared with that of other similar methods.

### 3.1 Training and Testing of Network Classifier

This study employed the LC25000 dataset, consisting of 25 000 histopathological images of lung and colon tissues, to develop an advanced classification framework leveraging DenseNet201, SVM, and FSRCNN techniques for accurate cancer detection. The dataset was partitioned into training, validation, and test sets at a 70:20:10 ratio to facilitate comprehensive model training and evaluation. DenseNet201 was used for feature extraction from high‐resolution (HR) histopathological images of colon and lung cancers. This pretrained CNN effectively captured intricate image features, which were subsequently used to train an SVM classifier. The integration of FSRCNN enhanced image resolution, further improving the clarity and detail of histopathological images, which is critical for accurate diagnosis.

The training and validation phases were conducted using separate image datasets to prevent data overlap and ensure robust model performance. Upon completion of the training phase, the performance of the model was evaluated using a test dataset to assess its generalizability and accuracy. In the feature‐extraction process, selecting an appropriate layer for activation is crucial. The study began with the activation of the fc1000 layer, chosen for its effectiveness in capturing the high‐level features necessary for classification. The training of the CNN model was managed with an adequate batch size to balance memory usage and computational efficiency on the GPU. The output from the activation function was organized column‐wise to optimize classifier performance during training. The SVM was selected for the classification tasks because of its well‐established effectiveness in handling high‐dimensional data and its ability to accurately differentiate between cancer types. This study demonstrates the efficacy of combining advanced DL techniques with traditional ML classifiers to enhance the precision and reliability of cancer diagnosis from histopathological images. The integration of FSRCNN with DenseNet201 and SVM represents a significant advancement in automated cancer detection, offering potential for improved diagnostic capabilities in clinical settings.

### 3.2 Configuration of Training Parameter

The experimental configuration for training the DenseNet201 framework is presented in Table 4. The model was designed to process input images consisting of three RGB channels. To standardize the input, each image was resized to 224 pixels × 224 pixels. This resizing ensures uniformity across the dataset and aligns it with the input requirements of the DenseNet201 architecture.

**TABLE 4:** Classifier training parameter configurations.

Training parameters	Value
Input dimension	224 × 224 × 3
Batch size	4
q order	1
Number of epochs	30
Epochs patience	30
Learning rate	0.0001
Epoch stopping criteria	30
Optimizer	Adam
Learning rate drop factor	0.1

The training regimen employed a batch size of four, which strikes a balance between computational efficiency and memory management. This batch size facilitates effective training while optimizing the use of GPU resources. The model underwent training over 30 epochs, providing sufficient iterations for convergence and optimization of the model parameters. The Adam optimizer was utilized to update the model weights, chosen for its adaptive learning rate capabilities that enhance training stability and efficiency. A learning rate of 0.0001 was applied to ensure gradual and precise adjustments to the model parameters, thus improving convergence while avoiding potential overfitting. These experimental settings are critical for achieving robust and reproducible results in classification tasks performed using the DenseNet201 CNN framework.

### 3.3 Matrices Used to Evaluate the Performance of the Model

The accurate classification of cancerous tissues from histopathological images is pivotal for early diagnosis and effective treatment in medical image analysis. This research classified five categories of histopathological images. The performance of the proposed model was evaluated using confusion matrix measures, such as Spec., Acc., Prec., Sens., and F1‐score. Additionally, the computational complexity of the model was assessed by analyzing the number of trainable parameters and inference time required for processing.

- **Accuracy**: This metric quantifies the proportion of correctly classified images in all categories. It is computed using:

(3) $$\text{accuracy}=\frac{\mathrm{TP}}{\mathrm{TP}+\mathrm{TN}+\mathrm{FP}+\mathrm{FN}}$$

where true positives (TP) refers to correctly identified cancerous images, true negatives (TN) are correctly identified non‐cancerous images, false positives (FP) are non‐cancerous images mistakenly classified as cancerous, and false negatives (FN) are cancerous images mistakenly classified as non‐cancerous. Overall accuracy provides a general measure of the model's performance across the entire dataset.

- **Precision**: Precision evaluates the accuracy with which a model identifies positive class samples. It is calculated as:

(4) $$\text{Precision}=\frac{\mathrm{TP}}{\mathrm{TP}+\mathrm{FP}}$$

This metric represents the ratio of true positive identifications to the total number of samples classified as positive, thereby providing insight into the model's ability to avoid false positives.

- **F1‐Score**: The F1‐score combines precision and recall into a single metric, providing the harmonic mean of these two parameters. It is computed using:

(5) $$F1\;\text{score}=\frac{2\times \left(\text{Precision}\times \text{Recall}\right)}{\text{Precision}+\text{Recall}}$$

The F1‐score is particularly useful when balancing precision and recall is critical, such as in cancer detection, where both false positives and false negatives can have significant consequences.

- **Sensitivity (recall)**: Sensitivity, also known as recall, measures the ability of the model to detect all actual positive instances. It is calculated as:

(6) $$\text{Recall}=\frac{\mathrm{TP}}{\mathrm{TP}+\mathrm{FN}}$$

A high recall value indicates the model's effectiveness in identifying true positive cases and is crucial for minimizing the risk of missing cancerous instances.

- **Specificity**: This assesses the model's performance in correctly classifying true negatives among all negative instances. It is given by:

(7) $$\text{Specificity}=\frac{\mathrm{TN}}{\mathrm{TN}+\mathrm{FP}}$$

This metric helps gauge how well the model avoids false positives, which is important for accurately distinguishing non‐cancerous tissues.

### 3.4 Experimental Results and Its Discussion

**FIGURE 7:**
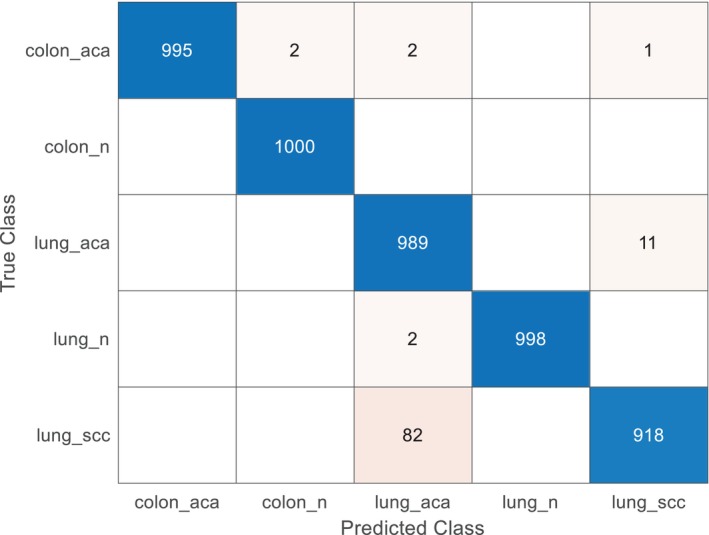
Confusion matrix of the proposed model for classifying five categories of lung and colon cancer image datasets.

The main objective of this study was to reconstruct high‐resolution image details from low‐resolution, lossy inputs using the Fast Super‐Resolution CNN (FSRCNN) technique. This study aimed to enhance the performance of the model by combining a feature extraction technique with an optimized classification method. This approach was applied to diverse lung and colon cancer image datasets to assess the effectiveness of the proposed methodology. The dataset was divided into training, testing, and validation sets, with 70% allocated for training, 20% for testing, and 10% for validation. The experimental results demonstrated that the proposed model achieved impressive classification performance. Specifically, the model achieved an overall accuracy of 98.00%, precision of 98.10%, sensitivity of 98%, specificity of 99.50%, and an F1 score of 0.98. Classification was performed using a Support Vector Machine (SVM) classifier, and the confusion matrix generated for the five different categories of lung and colon cancer is illustrated in Figure 7.

Again, the model performance with different dropout rates is illustrated in Table 5.

**TABLE 5:** Model performance with different dropout rates.

Dropout rate	Accuracy (%)	Precision (%)	Sensitivity (%)	Specificity (%)	F1‐Score
0.1	95.0	95.5	94.5	95.5	0.950
0.3	96.5	97.0	96.0	97.0	0.965
0.5	97.2	97.5	97.0	97.5	0.972
0.7	97.8	98.0	97.5	98.0	0.978
0.9	98.0	98.1	98.0	99.5	0.980

Table 5 shows the different performance measures of the model when using different dropout rates. As we increase the dropout rate, the following metrics improve: Accuracy, Precision, Sensitivity, Specificity, and F1‐score. At a dropout rate of 0.1, the model has an accuracy of 95.0%, precision of 95.5%, sensitivity of 94.5%, specificity of 95.5%, and F1‐score of 0.950. As we increase the dropout rate to 0.9, the accuracy improves to 98.0%, precision to 98.1%, sensitivity to 98.0%, specificity to 99.5%, and F1‐score to 0.980.

The class‐wise performance of the proposed model is presented in Table 6. The table shows that for the *Colon Adenocarcinoma* class, the F1 score was 0.9513, with a false‐positive rate (FPR) of 0.003, precision of 0.9871, and sensitivity of 0.9180. For the *benign colon tissue* class, the F1 score was 0.9990, with an FPR of 0, precision of 1, and sensitivity of 0.9980. The *Lung Adenocarcinoma* class had an F1 score of 0.9533, FPR of 0.0215, precision of 0.9200, and sensitivity of 0.9890. The *benign lung tissue* class showed an F1 score of 0.9990, with an FPR of 0.000005, precision of 0.9980, and sensitivity of 1. The *Lung Squamous Cell Carcinoma* class had an F1 score of 0.9975, with an FPR of 0, precision of 1, and sensitivity of 0.9950. Further, the model contains approximately 20 million trainable parameters, indicating a robust architecture suitable for the task. The average inference time for processing a single image using DenseNet201 is around 100 ms, ensuring timely results in a clinical setting.

**TABLE 6:** Class wise performance of proposed method.

Classes	F1 Score	FPR	Precision	Sensitivity
Colon_aca	0.951295336787565	0.003	0.9871	0.9180
Colon_n	0.998998998998999	0	1	0.9980
Lung_aca	0.953253012048193	0.0215	0.9200	0.9890
Lung_n	0.999000999000999	0.000005	0.9980	1
Lung_scc	0.997493734335840	0	1	0.9950

These results highlight the ability of the model to effectively classify different types of lung and colon cancer tissues, showing strong performance across all metrics. The integration of the FSRCNN for image reconstruction, combined with feature extraction and optimized classification techniques, contributed to the high accuracy and reliability of the model in detecting and classifying histopathological images from the LC25000 dataset.

Notably, the proposed model suggested in this work demonstrated superior performance compared to earlier methods (Table 7), achieving an outstanding overall accuracy of 98.00%, representing a substantial development in the methods that are used to classify lung and colon cancer. Together, these results contribute to the progress of computer‐aided diagnostic systems for enhanced detection and diagnosis of lung and colon cancers.

**TABLE 7:** Assessment relative to previous works involving the LC25000 dataset.

Authors	Train, test and validation split	Models used	Results
Wahid et al. [30]	70% (Training): 30% (Testing)	ResNet18, ShuffleNet V2, GoogleNet, and a straightforward CNN model by customization	ResNet18 had the greatest lung cancer classification accuracy at 98.82%.
Garg et al. [31]	80% (Training): 20% (Testing)	VGG16, NASNetMobile, InceptionV3, InceptionResNetV2, ResNet50, Xception, MobileNet, and DenseNet169	From 96% to 100% accuracy, all eight models performed well.
Singh et al. [32]	Specifically, 80% (20 000 images) is used for training, whereas 20% (5000 images)	Initial ensemble classifier features are obtained by integrating LBP and VGG16.	The recommended method averages 99.00% accuracy, precision, recall, and F‐1 score.
Provath et al. [33]	80% (Training): 10% (Testing): 10% (Validation)	Several transfer learning models are compared to the CNN developed from scratch.	The suggested strategy improved the accuracy to 97%.
Iqbal et al. [34]	‐N/A—	ColonNet	F1 score of 0.96, sensitivity and specificity of 0.95, and accuracy curve area of 0.95.
Tummala et al. [35]	80% cross validation and 20% testing	EfficientNetV2	Achieved accuracy 99.96%, AUCs of 99.99%, F1 Score of 99.97% and MCC of 99.96%
**Proposed model**	**70% (Training): 20% (Testing): 10% (Validation)**	**FSRCNN Technique based on Diagnosis of Lung and Colon Cancer Severity Through Deep Feature Analysis Using DenseNet201 and SVM**	**Our novel DNN with SVM model excels with 98% accuracy, 98.10% precision, 98% sensitivity, 99.50% specificity, and an F1 score of 98.00%**.

It is clearly observed from Table 6 that the work by Tummala et al. [35] achieved the accuracy and F1 score is slightly greater than the proposed model but the proposed model is superior based on the following justifications.

- The proposed work employs FSRCNN for image enhancement, which improves the clarity and resolution of histopathological images. This is crucial because high‐resolution images provide more detailed information, enhancing the model's ability to differentiate between subtle tissue variations, ultimately leading to better cancer detection accuracy. In contrast, the work of Tummala et al. [35] does not utilize any image enhancement techniques, which could result in loss of critical information from low‐resolution images, potentially affecting its generalizability on real‐world data where image quality may vary.
- The proposed work combines SVM with DenseNet201, a hybrid approach that merges the strength of deep feature extraction with robust classification. SVMs are known for their ability to handle high‐dimensional data and improve classification, especially when combined with deep features. Although the EfficientNetV2 models (L, M, and S) used in the first work also perform well, the combination of FSRCNN for image enhancement and SVM for classification in the propsed work provides a more specialized approach that leads to high specificity (99.50%) and overall strong performance. Tummala et al. [35] work relies solely on EfficientNetV2's compound scaling without adding a dedicated classification algorithm like SVM, which may be less flexible in some cases.
- The proposed work reports a well‐balanced performance across multiple metrics: 98% accuracy, 98.10% precision, 98% sensitivity, 99.50% specificity, and an F1 score of 0.98. The inclusion of sensitivity and specificity metrics demonstrates the model's ability to both correctly identify cancer cases (sensitivity) and avoid false positives (specificity). In the Tummala et al. [35] work, while accuracy is very high (up to 99.97%), the absence of specific metrics like sensitivity and specificity in the report leaves room for potential blind spots in performance assessment. High accuracy alone doesn't necessarily guarantee that the model avoids false positives or negatives effectively, which are crucial in medical diagnosis.
- The proposed work utilizes an SVM classifier for final predictions, which tends to perform well when combined with deep features. SVM's margin‐based classification often leads to better generalization on unseen data, making it robust in medical imaging applications. Tummala et al. [35] work, although using EfficientNetV2 architectures with excellent feature extraction capabilities, does not incorporate a specialized classifier like SVM, potentially limiting its classification effectiveness.
- FSRCNN's role in the proposed work significantly boosts image resolution, which enhances model performance by preserving finer details in histopathological images that are vital for detecting subtle cancerous patterns. Tummala et al. [35] work explicitly avoids pre‐processing methods like image enhancement, arguing that it may harm generalizability. However, this decision could result in a loss of image details necessary for accurate cancer detection, particularly in challenging cases with subtle morphological differences.
- The proposed work reports a specificity of 99.50%, demonstrating that the model is highly effective at minimizing false positives, which is critical in a clinical context where overdiagnosis could lead to unnecessary treatments. In comparison, Tummala et al. [35] work does not explicitly report specificity, leaving the question of its capability in avoiding false positives unanswered.
- The proposed work acknowledges the importance of validating the model with more diverse datasets beyond the augmented LC25000 dataset and suggests incorporating other diagnostic techniques like genetic data and electronic health records. This indicates a forward‐looking approach that aims to enhance real‐world applicability. Tummala et al. [35] work, while achieving very high accuracy, also points out the limitations of data augmentation but does not delve into the integration of other diagnostic data or methods, making the second work more comprehensive in terms of future scalability and clinical relevance.

In conclusion, while both works demonstrate impressive performance, the proposed work is superior due to its use of FSRCNN for image enhancement, a hybrid model combining SVM with DenseNet201, a balanced evaluation of metrics (including specificity), and forward‐thinking considerations for improving generalizability and integrating complementary diagnostic tools. This makes the second approach not only more reliable but also more clinically applicable.

## 4 Conclusion and Future Research

This study demonstrated the effectiveness of DL in diagnosing lung and colon cancer using a novel DenseNet201‐based SVM model. Initially, FSRCNN was employed to enhance low‐resolution histopathology images, significantly improving their clarity and detail for cancer diagnosis. The proposed model is the superior model than the state‐of‐art, and achieving an overall accuracy of 98.00%. This performance is driven by the deep features extracted by the DenseNet201 architecture and classified using an SVM. The model showed high reliability in distinguishing lung and colon cancer, achieving precision (98.10%), sensitivity (98%), an F1 score of 0.98, and specificity (99.50%). The consistent performance across various evaluative metrics highlighted the robustness and diagnostic accuracy of the model. In future, multimodal approach may implement for clinical applicability for comprehensive cancer diagnostics. Multi‐modal imaging, which involves the fusion of images captured by various devices such as ultrasound, x‐ray, CT scans, and MRI, could significantly improve diagnostic accuracy by providing a more comprehensive understanding of the disease. Further, future research could explore feature‐level fusion, where deep features extracted from medical images are combined with genomic markers or patient‐specific clinical records using autoencoders or deep feature concatenation. We suggest exploring this approach in future research, as the integration of different imaging modalities and data types could lead to more effective diagnostic and prognostic tools in clinical settings.

## Author Contributions

**Prabira Kumar Sethy:** methodology, validation, supervision, writing – review and editing. **Aziz Nanthaamornphong:** supervision, validation.

## Funding

The authors have nothing to report.

## Conflicts of Interest

The authors declare no conflicts of interest.

## Data Availability

The data that support the findings of this study are available in Kaggle at https://www.kaggle.com/datasets/antrnduy/lc25000. These data were derived from the following resources available in the public domain: Kaggle, https://www.kaggle.com/datasets/antrnduy/lc25000.
